# Choice of therapeutic interventions and outcomes for the treatment of infections caused by multidrug-resistant gram-negative pathogens: a systematic review

**DOI:** 10.1186/s13756-019-0624-1

**Published:** 2019-11-04

**Authors:** Sarah Melissa Nørgaard, Camilla Skaarup Jensen, Josefine Aalestrup, Christina M. J. E. Vandenbroucke-Grauls, Mark G. J. de Boer, Alma Becic Pedersen

**Affiliations:** 1 Department of Clinical Epidemiology, Aarhus University Hospital, Olof Palmes Allé 43-45, 8200 Aarhus, N Denmark; 2 Department of Infectious Diseases, Leiden University Medical Center, Albinusdreef 2, 2333 ZA Leiden, The Netherlands; 3 Medical Microbiology and Infection Control, Amsterdam University Medical Centers, Vrije Universiteit, De Boelelaan 1117 Amsterdam, 1081 HV Amsterdam, The Netherlands

**Keywords:** *A. baumannii*, Enterobacteriaceae, MDR bacteria, *P. aeruginosa*, Review, Treatment

## Abstract

**Background:**

Antimicrobial resistance is an increasingly serious threat to public health, and the increased occurrence of multidrug-resistant (MDR) bacteria is a concern in both high-income and low- and middle-income countries. The purpose of this systematic review was to identify and critically appraise current antimicrobial treatment options for infections with MDR Gram-negative bacteria.

**Methods:**

A literature search for treatment of MDR extended-spectrum beta-lactamase (ESBL)-producing Enterobacteriaceae*, A. baumannii*, and *P. aeruginosa* was conducted in MEDLINE in January 2019. Relevant studies published in English, German, and French that evaluated clinical success, microbiological success, and 30-day mortality outcomes were included. The population of interest was adult patients.

**Results:**

Of 672 studies, 43 met the inclusion criteria. Carbapenems are the most common antibiotics used for the treatment of ESBL-producing Enterobacteriaceae*.* The clinical and microbiological success was similar for group 1 carbapenems (imipenem, meropenem, or doripenem), group 2 carbapenems (ertapenem), and non-carbapenem antibiotics. Mortality data were contradictory for group 1 carbapenems compared to group 2 carbapenems. The most common treatment option for *A. baumannii* and *P. aeruginosa* infections was intravenous colistin, regardless of infection site. Clinical success and mortality were similar in *A. baumannii* infections treated with colistin combination therapy vs. colistin monotherapy, whereas heterogeneous results were found with respect to microbiological success. Monotherapy and colistin combination therapy were used against *P. aeruginosa* with clinical and microbiological success (70–100%) depending on the infection site and severity, and the antibiotic used. Ceftazidime-avibactam therapy for ESBL-producing Enterobacteriaceae and *P. aeruginosa* showed good clinical success in one study.

**Conclusion:**

We did not find robust evidence for antibiotic treatment of any infection with MDR Gram-negative bacteria, including ESBL-producing Enterobacteriaceae*, A. baumannii*, and *P. aeruginosa,* that would lead to a firm recommendation for one specific antibiotic over another or for monotherapy over combination therapy. The choice of antibiotic treatment should be based on susceptibility testing balancing the expected clinical success rate against the risk of development of antibiotic resistance and the risk of severe side effects.

## Background

Multidrug-resistant (MDR) infections constitute a serious public health problem [1] because they are difficult to treat effectively, leading to longer hospital stays, treatment failure, and adverse outcomes, such as complications and death [2, 3]. During the last few decades, several bacterial pathogens have evolved into MDR forms [1, 4]; of particular concern are MDR Gram-negative pathogens, such as Enterobacteriaceae, *Acinetobacter* spp., and *Pseudomonas aeruginosa* [5], which are becoming resistant to almost all available antibiotics [6]. Extended-spectrum beta-lactamase (ESBL)-producing Enterobacteriaceae are responsible for a variety of community-onset and healthcare-associated infections and are associated with poor clinical outcomes [7, 8]. *Acinetobacter baumannii* can cause a variety of infections, including pneumonia and bloodstream infections, which are associated with high mortality and morbidity [9, 10]. *P. aeruginosa* is one of the main causes of serious nosocomial infections in Europe, including pneumonia, bloodstream infections, and urinary tract infections [11]. Other MDR Gram-negative bacteria are emerging, but are still rare and not a focus of the current review.

The European Centre for Disease Prevention and Control estimated in 2019 that infections caused by a subset of resistant bacteria are responsible for approximately 33,000 deaths in Europe annually [12]. The overall crude economic burden of antibiotic resistance in Europe has been estimated to be at least 900 million Euro in health care costs and 600 million Euros a year in lost productivity [4, 13]. Despite the increased occurrence of MDR organisms, data on which antimicrobial treatment with a single antibiotic or a combination of two or more are scarce [14]. The available guidelines from the USA and Australia are based on data up to 2015, which may already be obsolete because of the expanding evolution of MDR Gram-negative bacteria. European guidelines mostly focus on preventative measures thought to reduce the occurrence of MDR Gram-negative bacteria [5, 15–20]. Previous systematic reviews were based on heterogeneous studies with small, diverse populations from single centers, comparing various antimicrobial treatment options, and providing different results.

The aim of this systematic review was to evaluate how different antimicrobial treatments used in adult patients against MDR infections, focusing on ESBL-producing Enterobacteriaceae, *A. baumannii*, and *P. aeruginosa*, affect clinical success and mortality outcomes.

## Methods

The review protocol was not registered with the international prospective register of systematic reviews.

### Eligibility criteria

Eligible study designs included randomized clinical trials, observational studies, prospective or retrospective design, concomitant or historical control studies, meta-analyses, and systematic reviews. Studies investigating any antimicrobial treatment for infections caused by MDR Gram-negative bacteria (ESBL-producing Enterobacteriaceae*, A. baumannii*, and *P. aeruginosa*) were included.

The population of interest was adult patients (age 18 years or older) who had a confirmed MDR infection and received antimicrobial treatment. We included studies that evaluated the outcomes of specific MDR Gram-negative bacteria with regard to the administered antimicrobial treatment. Studies directly comparing outcomes following different antibiotic treatments were of particular interest. However, we also included studies reporting the outcomes of specific treatments without a comparison treatment group.

The primary outcome of interest was clinical success from initiation of treatment until discharge or death. Clinical success was defined as complete resolution or substantial improvement of the signs and symptoms of the index infection, such that no further antibacterial therapy was necessary. Secondary outcomes were mortality, regardless of follow-up time after infection, or initiation of treatment and microbiological success measured by microbiological response, suppression, or eradication, bacteriological count, and laboratory outcome.

Studies published between January 1, 2006, and January 18, 2019, were included. For further details, please see the PICOS table (Additional file 1).

### Information source

MEDLINE was searched via the PubMed electronic database under the guidance of a research librarian for articles, and the reference lists of the included articles were reviewed to find additional articles.

### Literature search

Our search strategy included the following search terms: “multidrug resistant” AND “gram negative bacteria” AND “ESBL” OR “*A. baumannii*” OR “*P. aeruginosa*” (Additional file 2). We limited our search to the English, German, and French languages and studies in adult patients (≥18 years). The search terms covered the title and abstract of the paper. We included studies with any method of diagnosing MDR infection and any antimicrobial treatment. Many definitions have been used to characterize MDR infection, and most articles were not clear about the definition. The European Committee on Antimicrobial Susceptibility Testing (EUCAST) defines MDR as acquired non-susceptibility to at least one agent in three or more antimicrobial categories [21]. If the authors classified infection as MDR, then the article was eligible for review, as we can only assume the EUCAST definition was applied. Any site of infection was included, including the respiratory tract, bloodstream, and urinary tract. Studies were selected through a three-stage selection process described below. Additional articles were identified by checking the references of the already selected papers.

### Study selection

Our initial search targeted articles that 1) evaluated infections with MDR ESBL Enterobacteriaceae*, A. baumannii,* or *P. aeruginosa*, 2) mentioned a potential antimicrobial treatment, and 3) included information on the outcome of treatment. First, a literature search was performed independently by three reviewing authors (SMN, CSJ, and JA), selecting relevant papers with the aforementioned MDR Gram-negative bacteria included in the title. Second, abstracts were reviewed by three reviewing authors for the other two eligibility criteria (administered antimicrobial treatment and outcome of interest). Due to different nomenclature for MDR, after consulting with the senior tie-breaking author (ABP), we decided to include different synonyms (e.g., carbapenem resistance and extremely drug-resistant) in the study selection process to insure inclusion of all articles concerning MDR bacteria. At the third stage, the full-text versions of potentially eligible publications were obtained and distributed evenly between the three reviewing authors and examined in detail according to a predefined extraction form (Tables 1, 2 and 3). Standardized, pre-determined, study criteria were applied to all full-text documents.

**Table 1:**
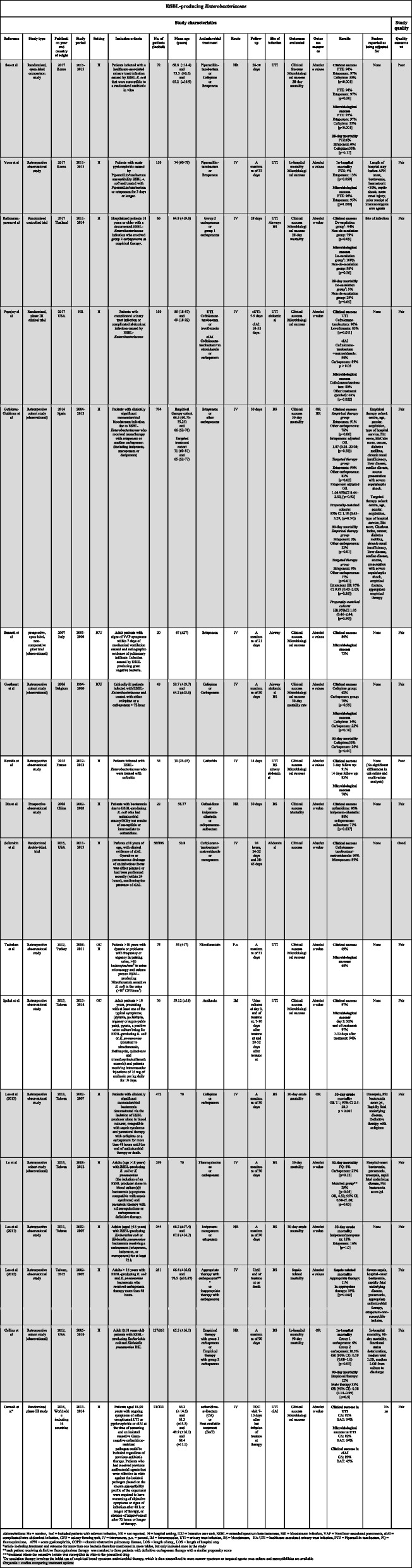
Choice of treatment and outcomes for ESBL-producing *Enterobacteriaceae*

**Table 2:**
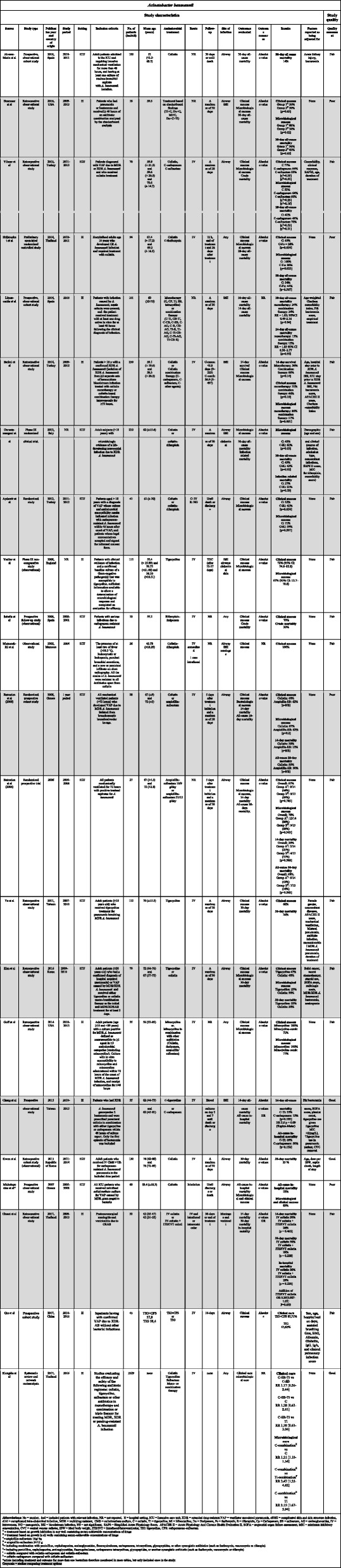
Choice of treatment and outcomes for *A. baumannii*

**Table 3:**
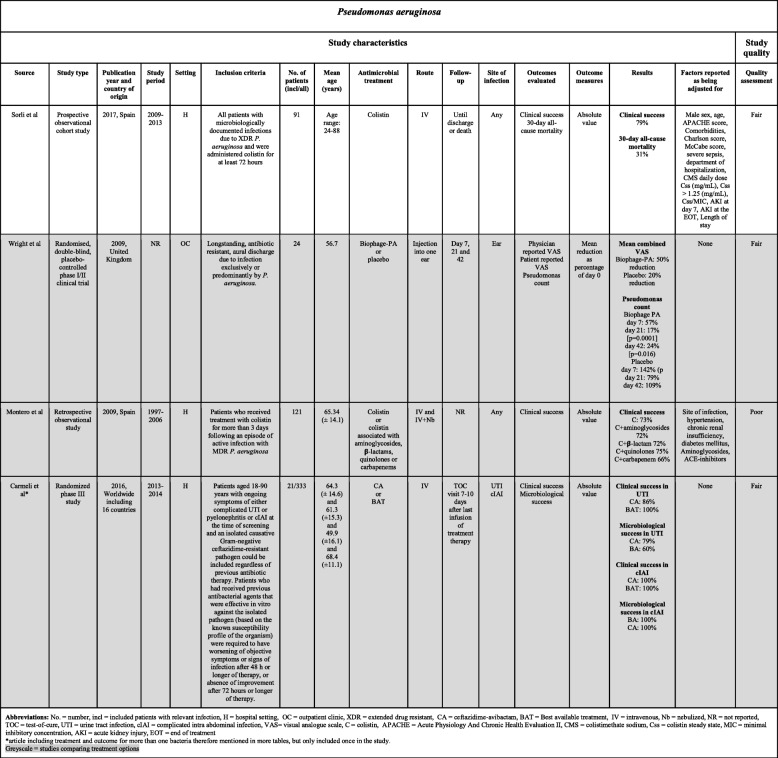
Choice of treatment and outcome for *Pseudomonas aeruginosa*

**Fig. 1:**
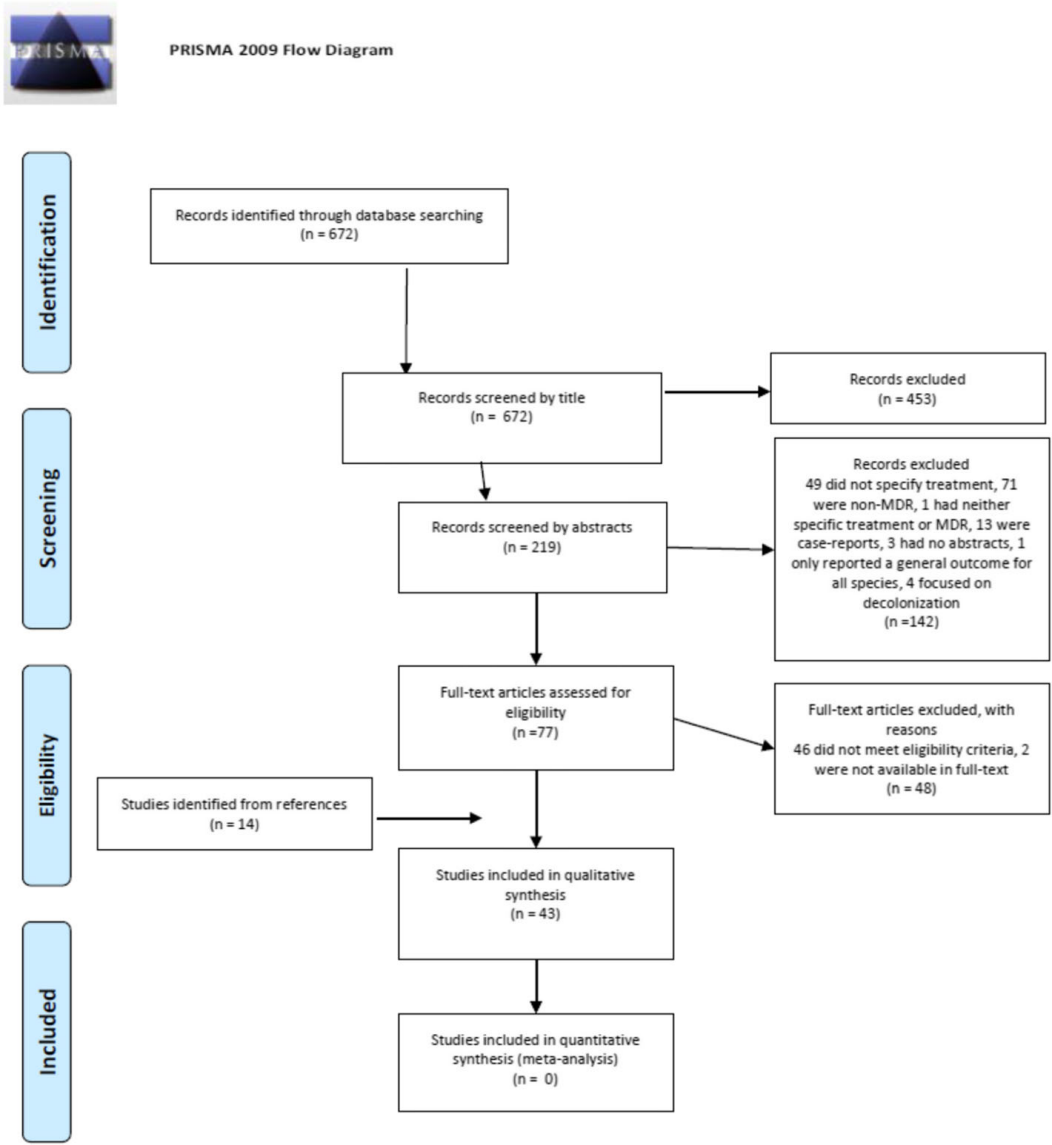
PRISMA flow diagram

At each stage, disagreements about the fulfilment of eligibility criteria were resolved by consensus or in consultation with the senior tie-breaking author. Search results were exported to EndNote V.X7.4 (Thomson Reuters, New York, New York, United States) and duplicates removed. The EndNote database with full-text articles is available upon request. The selection process is presented in Fig. 1.

### Data extraction and assessment of study quality

Data were extracted using a structured and standardized form piloted in six studies (Tables 1, 2 and 3). Discrepancies were compared to the original data. Information was extracted on the following characteristics: author names, year of publication, country of origin, study design, study period, characteristics of the study population (size, age, inclusion criteria, and site of infection), follow-up time, antimicrobial treatment and administration, outcome evaluated, factors for which the analysis was adjusted, statistical analyses, and risk estimates with *p*-values. Each review author presented extracted data for discussion with the other two review authors. If a review author had any doubt regarding extracted data, the paper was reviewed by another review author and disagreements resolved by discussion between the two review authors or in consultation with the senior author.

Quality and risk of bias in individual studies were assessed at the study and outcome level jointly by all reviewing authors using the Study Quality Assessment Tool from The National Heart, Lung, and Blood Institute [22]. The results of the quality assessment are presented in Tables 1, 2 and 3. Each study was quality rated according to one of the following categories based on the proportion of yes answers to all relevant questions: poor quality, 0–40%; fair quality, 41–80%; and good quality, 81–100% [22]. Quality assessments were conducted by the three authors jointly. Disagreements about the quality assessment were resolved by consensus or in consultation with the senior author.

### Summary measures

The following measures of treatment success were included: absolute values, absolute risk differences, hazard ratio (HR), relative risk, and odds ratio. Unadjusted and adjusted measures were included if available.

### Planned methods of analyses

The investigators considered quantifying effect measures in a weighted formal meta-analysis if there were consistency in the study designs, participants, antimicrobial treatment, and reported outcome measures. Otherwise, the systematic review would focus on describing the studies, their results, their applicability, and their limitations, and a qualitative synthesis of the results rather than a meta-analysis.

The systematic methodology of this review was based on the Preferred Reporting Items for Systematic Reviews and Meta-Analyses statement [23].

## Results

### Study selection

The literature search identified 672 studies. After an initial screening of the titles, 453 studies were excluded. Another 142 articles were excluded after reading the abstracts and 48 articles were excluded after reading the full-text because they did not meet the eligibility criteria. An additional 14 articles were included after identifying them from the reference lists of the already included papers. A total of 43 articles were included in the qualitative systematic review (Fig. 1).

### ESBL-producing Enterobacteriaceae

**Fig. 2:**
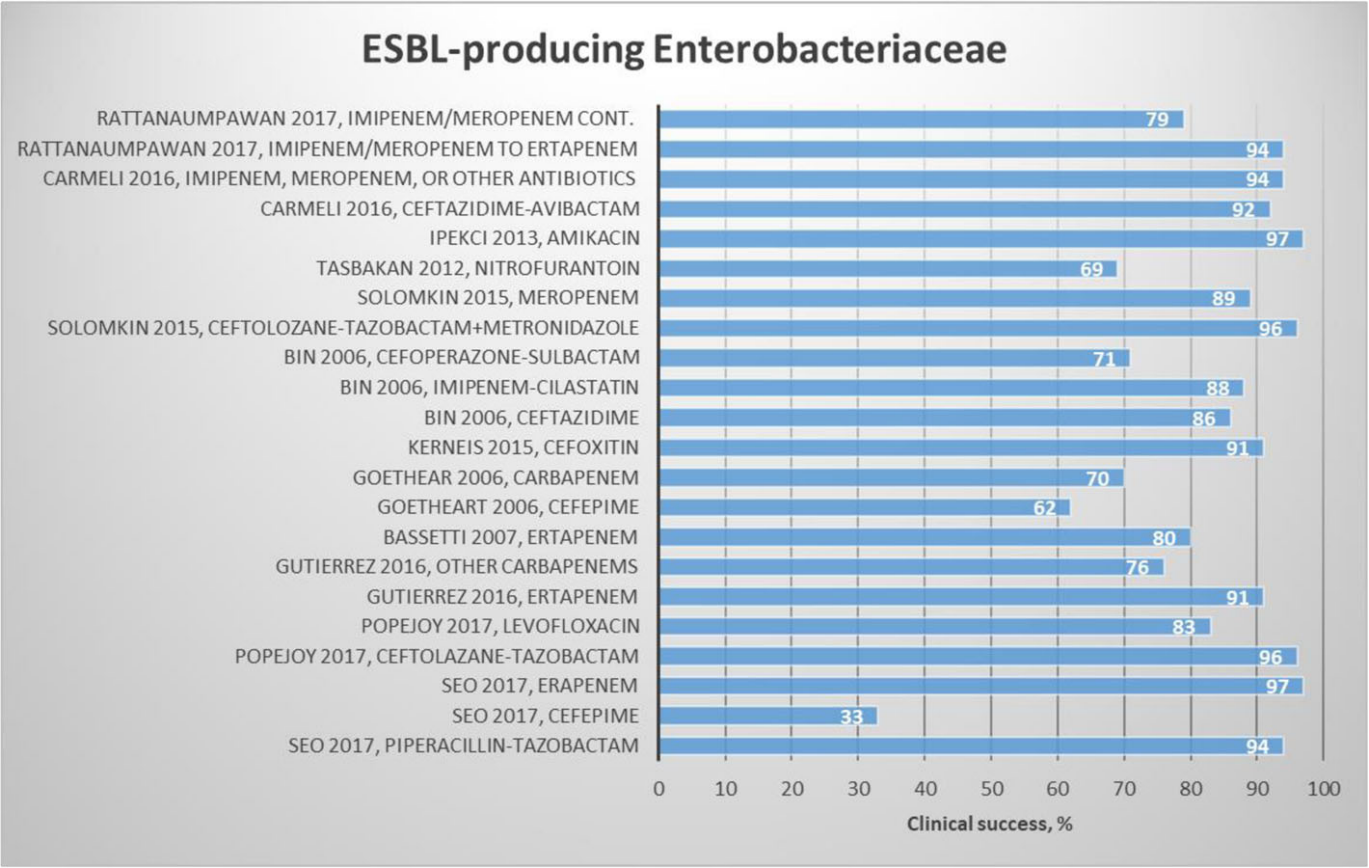
Results regarding choice of treatment and clinical success for ESBL-producing Enterobacteriaceae

**Fig. 3:**
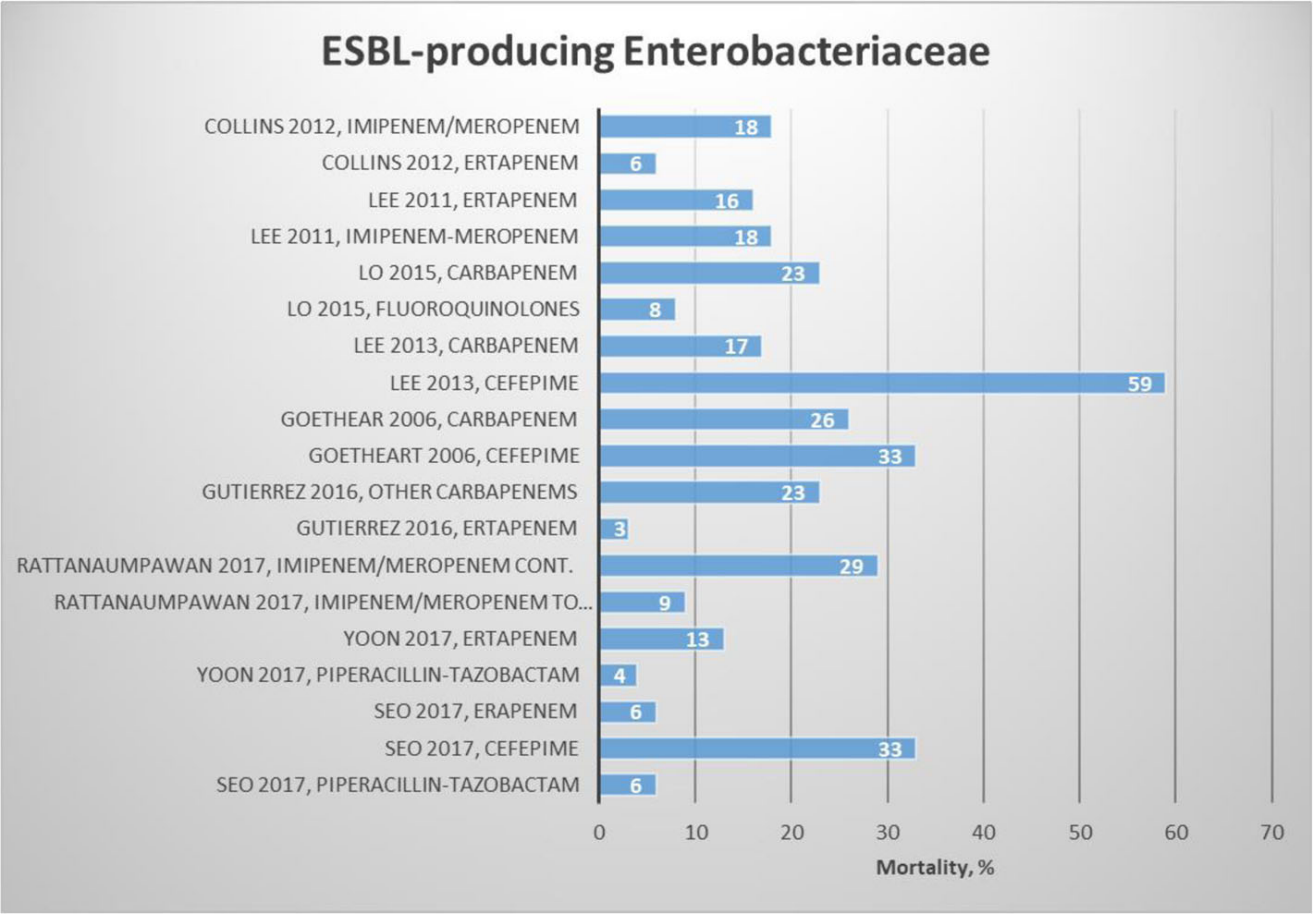
Results regarding choice of treatment and mortality for ESBL-producing Enterobacteriaceae

We included 18 articles: 13 observational studies [24–36] and 5 randomized studies [37–41] (Table 1). Clinical success and mortality by antibiotics are presented in Figs. 2 and 3. Four studies compared treatment with group 1 carbapenems (ertapenem) to treatment with group 2 carbapenems (imipenem/meropenem) [25, 32, 36, 38]. No difference was found in clinical and microbiological success [38], but conflicting results were reported for mortality [32, 36, 38].

Lee et al. reported a lower sepsis-related mortality among 251 patients receiving appropriate therapy (11%) compared to those receiving inappropriate therapy (38%) regardless of whether it was ertapenem, imipenem, or meropenem [33]. Bassetti et al. showed that treating ventilator-associated pneumonia with ertapenem had more than 75% clinical and microbiological success [42]. No difference was found in 30-day mortality for treatment with fluoroquinolones compared to carbapenems, whereas patients treated with cefepime were more likely to die within 30 days than patients treated with carbapenems [34]. A single study [24] found no difference between ertapenem and piperacillin-tazobactam in mortality or microbiological success for patients with acute pyelonephritis.

Goetheart et al. compared imipenem/meropenem as monotherapy or in combination with other antibiotic treatment options to treatment with cefepime [27]. Patients treated with cefepime and imipenem/meropenem had similar clinical and microbiological success and 30-day mortality. Solomkin et al. found no difference in clinical success between ceftolozane/tazobactam+metronidazole and meropenem treatment [40]. Carmeli et al. investigated ceftazidime-avibactam against group 2 carbapenem monotherapy (mostly imipenem and meropenem, but also other treatments) [41] in 263 patients with urinary tract infection and 20 patients with complicated intra-abdominal infections (cIAIs) caused by ESBL-producing Enterobacteriaceae or *P. aeruginosa*. Clinical success was reported in more than 92% of patients with urinary tract infection caused by ESBL-producing Enterobacteriaceae treated with ceftazidime-avibactam and best available treatment (monotherapy with amikacin, colistin, doripenem, ertapenem, ertapenem sodium, gentamicin, imipenem, or meropenem piperacillin/tazobactam and combination therapy with ciprofloxacin + meropenem or colistin + imipenem), whereas microbiological success was achieved in 64% of patients treated with ceftazidime-avibactam compared to 82% treated with best available treatment. Clinical success was reported in 5 of 11 patients with cIAI due to ESBL-producing Enterobacteriaceae treated with ceftazidime-avibactam compared to 8 of 9 patients treated with the best available treatment.

Other treatment options included piperacillin-tazobactam vs. ertapenem vs. cefepime [37], ceftolozane/tazobactam vs. levofloxacin and ceftolozane/tazobactam vs. ertapenem [39], and ceftazidime vs. imipenem/cilastatin vs. cefoperazone/sulbactam [29]. Seo et al. reported a difference in clinical success when treating 72 patients with urinary tract infection with piperacillin-tazobactam vs. ertapenem vs. cefepime (94% vs. 97% vs. 33%), whereas microbiological success and 28-day mortality were similar [37]. A phase III clinical trial [39] investigated patients with urinary tract infection randomly assigned to treatment with ceftolozane-tazobactam or levofloxacin, and patients with cIAI randomly assigned to treatment with ceftolozane-tazobactam or ertapenem. Better clinical success was noted when treating urinary tract infection with ceftolozane/tazobactam compared to levofloxacin (98 and 83%). The clinical success in patients with cIAI was 96% for ceftolozane/tazobactam and 89% for carbapenem. Bin et al. found similar clinical success when treating with ceftazidime imipenem/cilastatin and cefoperazone/sulbactam [29].

In conclusion, for ESBL-producing Enterobacteriaceae, treatment with carbapenems (ertapenem and meropenem) was associated with low sepsis-related mortality [33]. Seven studies found similar effects between a number of alternative treatment options and carbapenems regarding mortality [24, 27, 35, 37], clinical success [27, 40, 41, 43], and microbiological success [27, 43]. Regarding the clinical success, the following drugs alone or in combination had a success rate > 90%: piperacillin-tazobactam, ceftolazane-tazobactam, ertapenem, ertapenem, cefoxitin, ceftolozane-tazobactam in addition to metronidazole, amikacin, and cetazidime-avibactam. In addition, the following drugs had 80 to 90% clinical success: levofloxacin, ceftazidime, imipenem-cilastatin, and meropenem. Mortality was less than 10% for piperacillin-tazobactam and fluoroquinolones. However, more than 10% mortality was observed for cefepime, imipenem/meropenem, and carbapenem. Conflicting results regarding mortality were observed for ertapenem.

### Acinetobacter baumannii

We identified 22 studies, 16 of which were observational studies [44–59], five were randomized clinical trials [60–64], and one was systematic review and meta-analysis [65] (Table 2). The studies were based on populations with different sites of infection (airways, bloodstream, abdomen, skin, and meninges) and study population size varying from 10 to 250 patients.

Four articles compared colistin monotherapy to colistin combination therapy. Yilmaz et al. reported 77% clinical success with colistin monotherapy compared to 64 and 55% for colistin-carbapenem therapy and colistin-sulbactam therapy, respectively [46]. Sirijatupha et al. reported 63% clinical success with colistin monotherapy and 56% for colistin-fosfomycin combination therapy [60]. Batirel et al. reported a clinical success rate of 31% for monotherapy and 46% for colistin combination therapy (carbapenem, sulbactam, and other agents) [48]. Finally, Aydemir et al. reported 52% clinical success with colistin monotherapy compared to 41% with colistin-rifampicin [61].

Conflicting results have been reported regarding the microbiological success of colistin monotherapy compared to combination therapy with carbapenem or sulbactam. Two studies found no difference in microbiological success [46, 61], whereas Batirel et al. found that combination therapy for bloodstream infection had a better microbiological outcome than monotherapy [48]. Durante-Mangoni et al. found that microbiological success was more likely with colistin-rifampicin combination therapy than colistin monotherapy [64]. Systemic colistin and combinations with localized colistin have been shown to have similar effects on mortality [59].

Therapy with ampicillin-sulbactam was not superior to colistin monotherapy with regard to clinical success, 14-day mortality, or 28-day all-cause mortality among patients with MDR *A. baumannii* ventilator-associated pneumonia [62]. Betrosian et al. found similar clinical success and 30-day mortality when comparing low and high doses of ampicillin-sulbactam among patients with ventilator-associated pneumonia, but microbiological success was better in the low dose group [63]. Colistin-fosfomycin combination therapy had better microbiological success than colistin monotherapy, whereas 28-day all-cause mortality was similar when MDR *A. baumannii* infection at various sites was treated [60]. Tigecycline therapy was not superior to colistin therapy in terms of microbiological and clinical success or 30-day mortality among critically ill patients with MDR *A. baumannii* pneumonia [52, 58]. In another study, combination therapy with colistin-tigecycline and colistin-carbapenem resulted in 14-day all-cause mortality of 35% vs. 15% and all-cause in-hospital mortality of 69% vs. 50%, respectively [54]. Kengkla et al. reported similar clinical success when comparing different colistin combination therapies to different colistin monotherapies, but a better microbiological outcome was demonstrated with colistin combination therapy vs. colistin monotherapy, and tigecycline combination therapy vs. tigecycline monotherapy [65]. No difference was found in all-cause mortality between colistin combination therapy vs. sulbactam combination therapy [65]. Antibiotics other than colistin were evaluated in several small studies [49–51, 53, 57].

**Fig. 4:**
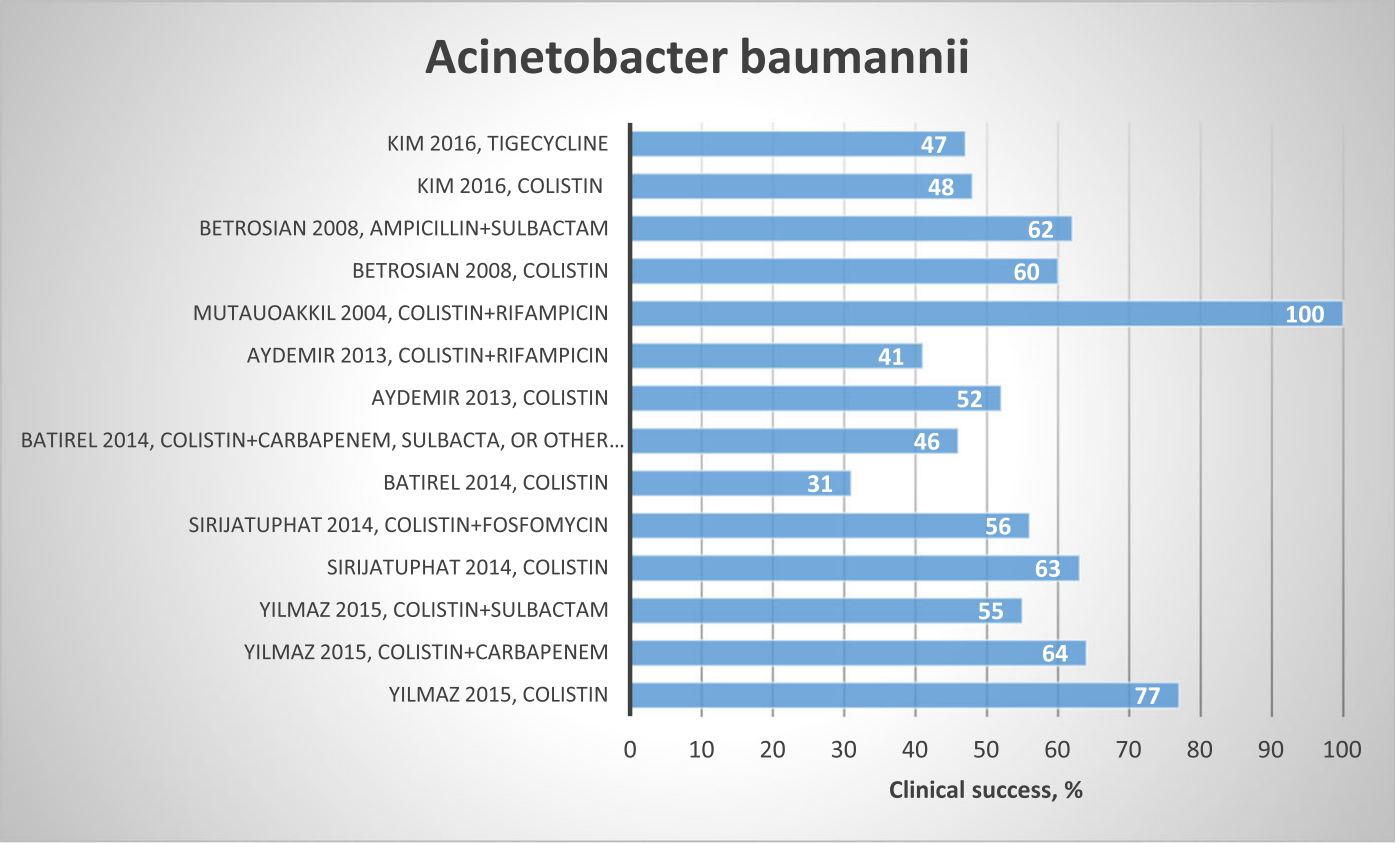
Results regarding choice of treatment and clinical success for *A. baumannii*

In conclusion, for *A. baumannii*, colistin combination therapy had no clear advantage over colistin monotherapy in regards to clinical success [46, 48, 60, 61] (Fig. 4). However, conflicting results have been reported regarding microbiological success when evaluating colistin monotherapy and colistin in combination with carbapenem [46], sulbactam [48], or rifampicin [61, 64]. Therapy with colistin monotherapy did not have a better outcome than ampicillin/sulbactam [52], and no difference in mortality was reported for any treatment comparison. Furthermore, tigecycline and minocycline [49, 51, 58] had a good effect on clinical and microbiological outcome, but the studies were small.

### *Pseudomonas aeruginosa*

**Fig. 5:**
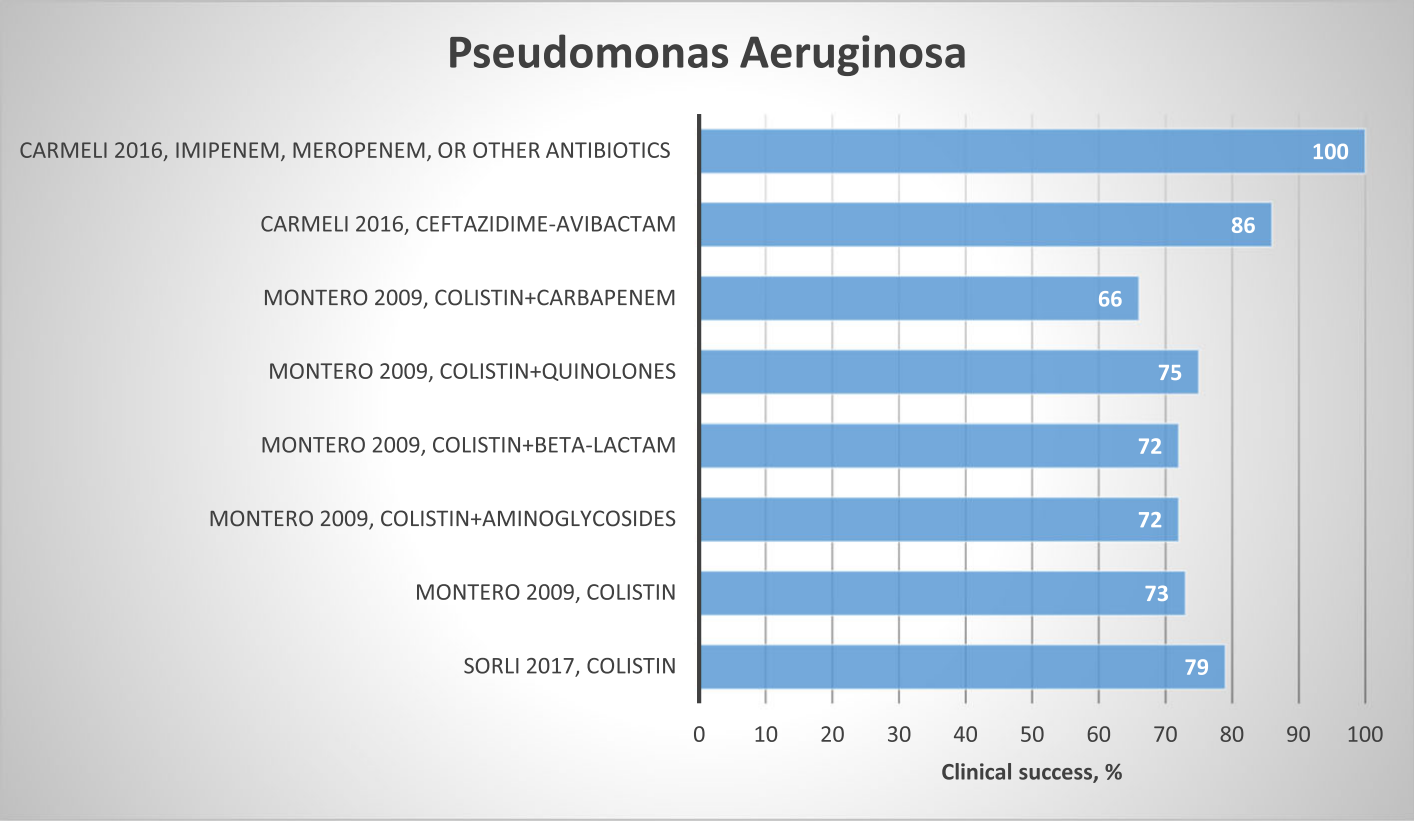
Results regarding choice of treatment and clinical success for *P. aeruginosa*

Four studies on *P. aeruginosa* were included in our review: two observational studies [66, 67] and two randomized controlled studies [41, 68] (Table 3). The largest study population comprised 263 patients. Clinical success rates are presented in Fig. 5.

Sorli et al. reported a clinical success rate of 79% after 30-days and a 30-day mortality of 31% for treatment with intravenous colistimethate sodium for *P. aeruginosa* infection at any site, except acute bronchitis and tracheitis [66]. Montero et al. reported similar clinical success for treatment with colistin monotherapy versus colistin combination therapy (aminoglycosides, β-lactams, quinolones, and carbapenems) in patients infected with *P. aeruginosa* at different infection sites [67]. Carmeli et al. compared ceftazidime-avibactam to the best available treatment (monotherapy with amikacin, colistin, doripenem, ertapenem, ertapenem sodium, gentamicin, imipenem, meropenem or piperacillin/tazobactam and combination therapy with ciprofloxacin + meropenem or colistin + imipenem) for patients with urinary tract infection or cIAI caused by MDR *P. aeruginosa* or ESBL-producing Enterobacteriaceae. Clinical and microbiological success occurred in 86 and 79% of patients with *P. aeruginosa-*specific urinary tract infection treated with ceftazidime-avibactam and 100% of patients treated with the best available treatment [41].

In conclusion, for *P. aeruginosa*, evaluated studies were characterized by heterogeneous study design, site of infection, and treatment used. Clinical success between 70 and 100% was reported regardless of the type of antibiotic treatment (Fig. 5). A high clinical success rate of up to 100% for ceftazidime-avibactam was demonstrated in the randomized study of Carmeli et al., in which a number of exclusion criteria were applied (i.e., both patients with complicated urinary tract infection and intra-abdominal infection were excluded, as were patients with evidence of abnormal liver function). Due to small sample sizes and variability in the type of antibiotics used, it is not possible to recommend one specific antibiotic over another.

### Risk of Bias

In general, the study quality varied, 16% (n = 7) of studies were poor in terms of quality, 74% (n = 32) were fair, and only 9% (n = 4) were good quality (Tables 1, 2 and 3).

## Discussion

In summary, we identified 43 articles that report on the clinical success, microbiological success, and/or mortality of different treatment options for the three most common MDR Gram-negative bacteria: ESBL-producing Enterobacteriaceae, *A. baumannii,* and *P. aeruginosa*. A variety of antimicrobial regimens have been used, but we did not find robust evidence that would lead to a firm recommendation of one specific antibiotic over another or for monotherapy over combination therapy with regard to efficacy in infections caused by these three different groups of MDR bacterial species. For the treatment of ESBL-producing Enterobacteriaceae, the most commonly used antibiotics were carbapenems. The effect of group 1 carbapenems (ertapenem) compared to group 2 carbapenems (imipenem, meropenem or doripenem) was heterogeneous with regard to reducing mortality, whereas the clinical and microbiological success were similar for group 1 and 2 carbapenems and other non-carbapenem antibiotics. Carbapenem should be used as a ‘last-line’ antibiotic, and other antibiotics should be used based on the antibiotic resistance profile. For treatment of MDR *A. baumannii,* intravenous colistin was used as the first drug of choice. Clinical success and mortality were similar in cases treated with colistin combination therapy or monotherapy, whereas heterogeneous results were found with regard to microbiological success. One study compared ampicillin/sulbactam to colistin monotherapy and found that patient groups had a similar outcome. The most common option for treatment of MDR *P. aeruginosa* infections was intravenous colistin, regardless of infection site.

Adverse reactions to the antibiotics were not a focus of this study but are an important aspect in the treatment of patients, as dosage adjustments must be considered and may have affected the results in this review in terms of clinical success, bacteriological success, and mortality. Another perspective is that patients infected with the studied bacteria are often critically ill, which makes it important to have extensive knowledge of the effects and side effects of the treatment of choice (e.g., the occurrence of nephrotoxicity due to colistin treatment makes colistin a less favorable choice than other antibiotics). Antimicrobial therapy can contribute to curation, but in many complicated infections, surgery and drainage procedures are essential. The included studies that reported multivariate analyses often emphasized the confounding effects of the severity of illness and patient comorbidity.

Ceftazidime-avibactam was introduced in the USA in 2015 and on the European market in 2016 for treatment of adults with complicated urinary tract infections, complicated intra-abdominal infections, hospital-acquired pneumonia, and other infections caused by Gram-negative organisms in patients with limited treatment options [69]. Current evidence of the effectiveness of ceftazidime-avibactam compared to treatment with carbapenem monotherapy in patients with ceftazidime-resistant Enterobacteriaceae and *P. aeruginosa* is good, and combinations could be considered to reduce the occurrence of carbapenem-resistant bacteria.

To the best of our knowledge, no other systematic reviews have resulted in specific guidelines for treatment of MDR Gram-negative infections. A prior systematic review suggested that colistin combination therapy may be preferred to colistin monotherapy for severely ill patients infected with MDR *A. baumannii,* but no firm evidence could be found [19]. Another systematic review proposed treating carbapenem-resistant ESBL-producing Enterobacteriaceae and *P. aeruginosa* with carbapenem plus either colistin or tigecycline combination therapy in low-level resistant infections and colistin-tigecycline combination therapy in high-level resistant infections [18]. Similar findings were published by Rafailidis et al. in 2014, concluding that carbapenem in combination with colistin or high-dose tigecycline or aminoglycosides could be used for treatment of carbapenem-resistant ESBL-producing Enterobacteriaceae in cases in which the minimum inhibitory concentration ranges of carbapenems are ≤8 mg/L [20].

The included studies were heterogeneous in terms of study design, patient population, site of infection, choice of antibiotic treatment, duration of follow-up, and outcome definitions, making it difficult to compare the different treatments and combinations of antibiotics used. Subsequently, we were not able to pool results for a meta-analysis. Most patients included in the studies were critically ill, with multiple comorbidities, and admitted to an intensive care unit; these factors may lead to underestimating the specific effect of a certain antibiotic treatment on mortality. Some studies included patients regardless of the site of infection, whereas other studies included patients with specific infections, such as pneumonia or urinary tract infection. The severity of these infections is different, which again can affect the antibiotic treatment-related outcome. In addition, the studies were often based on small sample sizes, reducing the ability to find any effect difference and to consider confounder adjustment and multivariate regression analysis. Only a few studies [24, 25, 34, 36, 40, 44, 47, 51, 52, 54, 55, 64] presented a sample size estimation and adhered to it.

Our study has several limitations. We only used the MEDLINE database for the literature search, which may not cover all published articles. We limited our search to the English, German, and French languages. As countries speaking other languages may have greater problems with MDR bacteria, we may have missed articles published in other languages. However, due to the major shift towards the publication of studies in English, the extent and effects of language bias may have decreased over the last few years. Lack of a standard definition of MDR results in a great diversity of published papers when defining MDR [70]. Consequently, the use of the term MDR in our search strategy may not cover the same bacteria and drug resistance, and we may have missed some relevant articles. In an attempt to avoid excluding relevant literature, different synonyms were accepted as MDR (e.g., carbapenem resistance and XDR) and all references in the included articles were screened for eligibility. Our inclusion criteria did not take susceptibility profile testing into consideration. Therefore, our results do not differentiate between studies with adequate and inadequate empiric treatment based on the susceptibility profile and studies in which treatment was targeted after the microbiological results were available. However, the vast majority of studies did not clearly state whether the susceptibility profile testing was done before the initiation of treatment. Risk of publication bias is another limitation of this review. It is possible that studies reporting on antibiotic treatment with high clinical and microbiological success rates are more likely to be published. Approximately 50% of the studies are estimated to be unpublished, including a majority of studies with less significant or negative results. Furthermore, 36% of the included studies were found by screening the reference lists of published articles, which may have caused notation bias.

## Conclusions

A variety of antimicrobial therapies have been used for treatment of the three most common MDR Gram-negative bacteria: ESBL-producing Enterobacteriaceae*, A. baumannii,* and *P. aeruginosa*. Carbapenems, in many situations, may have similar clinical and microbiological success rates as other antimicrobial regimens when used for the treatment of infections caused by ESBL-producing Enterobacteriaceae. For treatment of MDR *A. baumannii,* clinical success and mortality were similar in cases treated with colistin combination therapy compared to monotherapy, as well as in several studies comparing colistin with other antibiotics. The most common choice for treatment of MDR *P. aeruginosa* infections was intravenous colistin, regardless of infection site. Other antibiotic therapies had a similar effect as colistin, but due to small sample sizes and variability in the type of antibiotics used, it is not possible to recommend one specific antimicrobial regimen over another. The choice of definite antibiotic treatment should be based on susceptibility testing balancing the expected clinical success rate against the risk of development of antibiotic resistance and the risk of severe side effects. Taking into account the absence of evidence and all considerations above, for now, a personalized medicine approach and involvement of specialists in infectious diseases and microbiology are key measures to provide optimal treatment for each patient affected by infection caused by MDR microorganisms.

## Supplementary information

**Additional file 1.** PICOS criteria.

**Additional file 2.** Search criteria.

## Data Availability

The datasets generated during the current study are available from the corresponding author upon reasonable request.

## Abbreviations

cIAI: Complicated intra-abdominal infection
ESBL: Extended-spectrum beta-lactamase
HR: Hazard ratio
MDR: Multidrug-resistant
