# Supplementary material for: Choice of therapeutic interventions and outcomes for the treatment of infections caused by multidrug-resistant gram-negative pathogens: a systematic review
Source: Antimicrob Resist Infect Control. 2019 Nov 4;8:170. doi: 10.1186/s13756-019-0624-1 (PMC6830003; doi:10.1186/s13756-019-0624-1)
Supplement: Supplementary file 1 — Additional file 1. PICOS criteria. [file 13756_2019_624_MOESM1_ESM.docx]

Supplement 1: PICOS

|  | Inclusion criteria | Exclusion criteria |
| --- | --- | --- |
| Population | Adults patients (age 18 years or older) who had a confirmed MDR infection and received antimicrobial treatment. Patients with infections caused by MDR Gram-negative bacteria (ESBL-producing Enterobacteriaceae, A. baumannii, and P. aeruginosa) | Patients younger than 18 years of age  No MDR Gram-negative bacteria (ESBL-producing Enterobacteriaceae, A. baumannii, and P. aeruginosa) infection |
| Intervention | Any antimicrobial treatment | None |
| Comparators | Not applicable | Not applicable |
| Outcome measures | Primary:  Clinical success (Defined as complete resolution or substantial improvement in the signs and symptoms of the index infection, such that no further antibacterial therapy was necessary from initiation of treatment until discharge or death).  Secondary:  Mortality, regardless of follow-up time after infection, or initiation of treatment.  Microbiological success measured by microbiological response, suppression or eradication, bacteriological count, and laboratory outcome. | Outcomes other than clinical success, mortality and microbiological success.  Outcome that did not distinguish between different microbes |
| Study design | Randomized clinical trials, observational studies, prospective or retrospective design, concomitant or historical control studies, meta-analyses, and systematic reviews | Case reports |
| Restrictions | English, German, and French languages  Publication date: 2006-2019 | Duplicates, abstract or full text not available, non-English studies |
