# Supplementary material for: Choice of therapeutic interventions and outcomes for the treatment of infections caused by multidrug-resistant gram-negative pathogens: a systematic review
Source: Antimicrob Resist Infect Control. 2019 Nov 4;8:170. doi: 10.1186/s13756-019-0624-1 (PMC6830003; doi:10.1186/s13756-019-0624-1)
Supplement: Supplementary file 2 — Additional file 2. Search criteria. [file 13756_2019_624_MOESM2_ESM.docx]

**Supplement 2**

Full search strategy:

((((((multidrug[All Fields] AND resistant[All Fields]) AND ("gramme negative bacteria"[All Fields] OR "gram-negative bacteria"[MeSH Terms] OR ("gram-negative"[All Fields] AND "bacteria"[All Fields]) OR "gram-negative bacteria"[All Fields] OR ("gram"[All Fields] AND "negative"[All Fields] AND "bacteria"[All Fields]) OR "gram negative bacteria"[All Fields])) AND ("escherichia coli"[MeSH Terms] OR ("escherichia"[All Fields] AND "coli"[All Fields]) OR "escherichia coli"[All Fields])) OR ("pseudomonas aeruginosa"[MeSH Terms] OR ("pseudomonas"[All Fields] AND "aeruginosa"[All Fields]) OR "pseudomonas aeruginosa"[All Fields])) OR ("acinetobacter baumannii"[MeSH Terms] OR ("acinetobacter"[All Fields] AND "baumannii"[All Fields]) OR "acinetobacter baumannii"[All Fields])) OR ("stenotrophomonas maltophilia"[MeSH Terms] OR ("stenotrophomonas"[All Fields] AND "maltophilia"[All Fields]) OR "stenotrophomonas maltophilia"[All Fields])) OR ESBL[All Fields] AND ((Clinical Trial[ptyp] OR Controlled Clinical Trial[ptyp] OR Guideline[ptyp] OR Meta-Analysis[ptyp] OR Observational Study[ptyp] OR Randomized Controlled Trial[ptyp] OR Review[ptyp] OR systematic[sb]) AND ("2017/08/01"[PDAT] : "2019/01/18"[PDAT]) AND "humans"[MeSH Terms] AND English[lang] AND "adult"[MeSH Terms])
